# “It was my own decision”: the transformational shift that influences a woman's decision to use contraceptives covertly

**DOI:** 10.1186/s12905-022-01731-z

**Published:** 2022-05-02

**Authors:** Jenna Hoyt, Jessie K. Hamon, Shari Krishnaratne, Emmanuel Houndekon, Dora Curry, Miriam Mbembe, Seth Marcus, Misozi Kambanje, Shannon Pryor, Ariko Angela Barbra, Herbert Muhumuza, Nathaly Spilotros, Jayne Webster

**Affiliations:** 1 Department of Disease Control, Faculty of Infectious and Tropical Diseases, London School of Hygiene & Tropical Medicine, London, UK; 2 Care Benin, Cotonou, Benin Cotonou, Benin; 3 Care USA, Atlanta, USA; 4 World Vision International, Nairobi, Kenya; 5 World Vision US, Monrovia, CA USA; 6 Save the Children International, Blantyre, Malawi; 7 Save the Children US, Washington, DC USA; 8 International Rescue Committee Uganda, Kampala, Uganda; 9 International Rescue Committee US, New York, USA

**Keywords:** Family planning, Contraception, Covert use, Women, Empowerment, Decision-making, Integration, Childhood immunisations

## Abstract

**Background:**

Family planning (FP) is an important element of sexual and reproductive health and rights, but socio-cultural barriers and unbalanced gender relations often limit women’s decision-making about contraceptive use. Covert contraceptive use (CCU) exemplifies the limits on women’s decision-making and represents a way in which some women overcome constraints to achieve their reproductive goals. This study explores the decision-making process through which women choose to use contraceptives covertly.

**Methods:**

A qualitative synthesis was conducted using data from women, health providers, community members, health administrators, and intervention implementers (n = 400) to explore the decision-making process through which women choose to use contraceptives covertly. Interviews and focus group discussions were conducted at two time points as part of an evaluation of interventions integrating FP and childhood immunisation services at sites in Benin, Kenya, Malawi and Uganda. The sexual and reproductive health empowerment framework by Karp et al. (2020) was adapted and used to guide the analysis.

**Results:**

Women recognised that although they suffered the negative consequences of frequent pregnancies and of raising large families, they lacked overt decision-making power over their fertility. Women were confident to engage in CCU because they believed their husbands did not understand these consequences nor acknowledged their suffering, which justified not informing them. CCU was a difficult choice however, women felt comfortable voicing their reproductive preferences in settings where health providers were supportive.

**Conclusions:**

Women chose to use contraceptives covertly when they questioned the unfairness of their situation and recognised their own power to act in accordance with their reproductive preferences. This represented an important shift in a woman’s perception of who is entitled to make decisions about contraceptive use. Importantly, health providers can play a key role in supporting women’s autonomous decision making about contraceptive use and should be careful not to undermine women’s confidence.

## Background

Family planning (FP) is an important element of sexual and reproductive health (SRH) and rights and enables women to pursue their reproductive goals [1]. Though substantial gains have been made in improving access to FP services in low- and middle- income countries, women continue to face socio-cultural barriers when seeking to delay or stop childbearing [2]. Unequal gender-based power relations have emerged as a key constraint on woman’s reproductive health decision-making [3]. In particular, male reproductive preferences and disapproval of FP have been found to drive reproductive outcomes, including the use of modern contraceptive methods and family size [4–6]. To overcome these constraints, some women choose to engage in covert contraceptive use (CCU), that is contraceptive use without their husband’s knowledge. Previous estimates using data from three countries in sub-Saharan Africa (SSA) suggest that CCU represents between 6 and 20% of all contraceptive use [7]. More recent studies measuring CCU indicate that it remains a common way in which women circumvent their limited decision-making authority within relationships [5, 8, 9], and that in some countries the practice is growing [10]. Current literature on CCU in SSA explores women’s reasons for, and challenges with, using a contraceptive method covertly [5, 7–9]. In particular, findings highlight (1) women’s limited decision-making ability within a relationship; (2) male disapproval of contraceptive use, frequently linked to the fear of losing power over their wives; (3) the challenges of accessing discreet methods and concealing side effects and (4) the consequences of CCU being discovered. However, it has recently been highlighted that the decision-making process through which women choose to use contraceptives covertly has not been adequately explored [11].

CCU occupies a small but growing space within the wider discussion of women’s SRH empowerment and contraceptive use. CCU is often an indication that women lack decision-making power within their relationship [7]. However, it also represents a deliberate action by a woman to exert autonomy over her body and control her fertility [12]. What is less clear is what this practice means in the context of SRH empowerment. CCU is an example of a gender accommodating practice—one that evades harmful gender norms but does not address them. And yet, it has been noted that accommodating practices can play a role in challenging gender inequality [13]. But with the aim of making contraceptive use easier for women to take up and use overtly, gender transformative interventions—interventions that challenge gender norms and help shift community perceptions around FP and contraceptive use—are used by FP programme designers and implementers as a means of shifting community perceptions over the longer term [2, 5]. Engaging with men and including them in the FP sphere has also showed promise for increasing contraceptive uptake [14]. But it has been recently highlighted that whilst activities that improve men’s knowledge about FP and contraceptive use may help expand acceptance, direct male involvement in decision-making could have negative consequences for some girls and women [15]. Crucially, in a context where men still represent a significant obstacle to contraceptive use and the practice of CCU is prevalent, it is important for FP programmes to deliver services that respect women’s autonomy around contraceptive use. This tension is highlighted in the literature [5, 16, 17], and yet little is known about how FP programmes account for and deal with the practice of CCU. In light of these knowledge gaps, the primary aim of this study was to examine the process involved in women’s decisions to use contraceptives covertly, and specifically to explore this decision in the context of women’s SRH empowerment. In addition, this study offers insights to help programme designers and implementers support women who need to make autonomous decisions about contraceptive use.

## Methods

### Study design & approach

This study is a qualitative synthesis of empirical data from semi-structured interviews (SSIs), in-depth interviews (IDIs) and focus group discussions (FGDs) collected as part of a multi-country evaluation of the delivery of integrated FP and childhood immunisation services. It follows inductively from the primary analysis of the data, in that the thematic patterns that emerged around CCU were initially examined within their own country context. Findings from the primary analysis revealed striking similarities in the experiences of women engaging in CCU across these varied contexts. As such, these themes were subsequently appraised across the different contexts and countries and ultimately synthesised using an adaptation of Karp’s SRH framework [12]. The researchers recognise the importance of exploring the uniqueness of the contexts within which women make decisions around CCU. However, rather than examining the variations between the different contexts, this study set out to explore the common narratives across countries that emerged from the data.

### Study setting

The integrated FP and immunisation services were implemented over two phases at government health facilities with support from non-governmental organisations (NGOs) in Benin (CARE), Kenya (World Vision), Malawi (Save the Children) and Uganda (International Rescue Committee). The scale of the intervention varied by country and the number of sites increased between the first and second phase. The intervention sites were in predominantly rural areas and by the end of phase 2 included 30 health centres in Adjohoun-Bonou-Dangbo health zone, Ouémé Department in Benin; 34 health facilities in Garbatulla, Isiolo, Pokot South and Pokot Central in Kenya; 91 routine outreach clinics in Blantyre, Mwanza and Thyolo districts in Malawi; and 17 health centres in Karamoja sub-region in Uganda.

Intervention monitoring data relevant to the time period of the first evaluation indicated that discreet contraceptive methods (implants and injectables) made up a substantial proportion of the methods selected by new users (Benin 76.7%; Kenya 55.2%; Malawi 94.6%, Uganda 32.7%). As described in Webster et al*.* [18], there were important contextual factors that characterised the sites where the integrated services were delivered. In Benin’s study sites, the use of contraceptives was highly stigmatised within communities, in part due to a lack of male support, resulting in women not wanting to be seen accessing FP services for fear of stigmatization and judgement. In Kenya’s study sites, contraceptive use was seen as incongruous with religious values and the traditional desire for large families. In Malawi, the routine outreach clinics provided contraceptives to communities where health services were difficult to access but contraceptive use was generally supported within communities [19]. In Uganda’s study sites, contraceptive uptake was hampered by pervasive myths about contraceptive use and concerns about side effects. An additional contextual factor in Uganda was the high level of food insecurity in the area and the distribution of food rations at some health facilities, which influenced where women sought services [20].

Data from national Demographic and Health Surveys (DHS) reflect the variations in modern contraceptive use, unmet need for FP and women’s decision-making across the sites (Table 1). The regions are based on the administrative levels provided in the DHS and represent either the exact region where the intervention was located or the wider area that encompasses the site.

**Table 1:** Contraceptive use, women’s decision making and unmet need for FP by study site

	Benin [21] Ouémé	Kenya [22] Eastern /Rift valley	Malawi [23] Southern	Uganda [24] Karamoja
Modern contraceptive use				
Married women 15–49 years	15.2%	63.9%/46.8%	54.4%	6.5%
Decisions about contraceptive use among modern contraceptive users				
By woman only	57.2%	n/a	15.7%	27.7%
Jointly with husband	35.0%	n/a	75.3%	71.2%
Total	92.2%	n/a	91.0%	98.9%
Decisions about contraceptive use among non-users of modern contraceptives				
By woman only	75.7%	n/a	36.1%	28.8%
Jointly with husband	19.2%	n/a	50.5%	59.6%
Total	94.9%	n/a	86.6%	88.4%
Unmet need for FP				
Women reporting unmet need for FP	33.7%	12.4/20.8%	20.3%	19.7%

Modern contraceptives include Lactational amenorrhea (LAM)

### Intervention

The aim of the intervention was to improve access, uptake and use of modern contraceptive methods among postpartum women by integrating FP with childhood immunisation services. The integrated service delivery models varied by site but broadly involved the deliberate integration of services, whereby FP messaging and counselling was delivered during childhood immunisations at health facilities (Benin, Kenya, Uganda) or during routine outreach clinics alongside childhood immunisations and growth monitoring services (Malawi). Women interested in using a contraceptive were then referred from the immunisation (or growth monitoring) services to receive additional counselling on, and information about, FP and were provided the opportunity to take up a contraceptive method. Both FP and immunisation services were delivered on the same day at the same location, although not always at the same time. Additionally, in most sites the services were delivered by different health providers. At all sites, existing community structures and actors were leveraged to spread information about FP and to mobilise women to attend the integrated services. Actors included community health workers, community leaders, peer influencers (expert clients, male champions) and religious leaders (in Kenya). The type and availability of contraceptives varied by site but included a mix of condoms, oral contractive pill, injectables, implants and the intra-uterine device. In Uganda, fertility awareness-based methods were emphasised during counselling, particularly at the few faith-based facilities included in the study.

### Data collection

Qualitative data were collected at two different time points, the first round during the first phase of project implementation and the second round during the second phase. In the first round of data collection 71 SSIs and 21 FGDs with a total of 230 participants were conducted between October 2017 and March 2018. In the second round of data collection, 170 IDIs were conducted between June and November 2019. During both data collection rounds, purposive sampling was used for SSIs, IDIs and FGDs to select participants directly involved in the delivery or use of the integrated services or who had a role, or interest in, the design of the intervention. This included women (self-reported contraceptive users and non-users) of reproductive age, health providers (community- and facility- based), community leaders, peer influencers, male community members, religious leaders, health administrators and staff from the implementing NGOs. Participants were selected in consultation with the local partners in each country. An initial programme theory that reflected perceptions about what drove the success of the interventions as well as identifying the barriers was developed for each country and used to guide the participant selection process [25]. Maximum variation sampling was applied amongst the different categories of participants [26]. Women included in the study were attending the facility or outreach clinic to receive either or both immunisation and FP services. Health providers were selected based on their role delivering FP and/or immunisation services in facilities and clinics where implementing NGOs suggested the intervention was more or less well received. All participants were approached at the delivery site and provided consent to participate in the study. In the first round of data collection, SSIs and FGDs were conducted by SK and a trained local interviewer. In the second round, IDIs were conducted by a team of trained data collectors in each country.

During both data collection rounds, interviews were conducted on site and only the interviewers and participant were present. FGDs were conducted on site or in an outdoor community setting. In Benin interviews were conducted in French, Fon and Ouémé; in Kenya in Borana, Pokot and English; in Malawi in Chichewa and English; and in Uganda in Karamojong and English. Interviews and discussions were audio recorded and transcribed verbatim. When necessary, transcripts were transcribed and then translated into English.

Topic guides developed for the SSIs and FGDs were informed by the initial programme theories and adapted based on feedback from the implementing teams. In the first round of data collection, topics for health providers covered: workload, health care access, delivery of integrated services, socio-cultural norms and perceptions of women’s use or non-use of contraceptives. For women, topics included: reason for contraceptive use or non-use, barriers to contraceptive use, access to FP services and community acceptance of FP/contraceptive use. With other stakeholders, a mix of these topics was used depending on relevance to their role. A mix of SSIs and FGDs were employed, when feasible, to enable both a deeper exploration of the sensitive topics relating to FP and to facilitate rich discussions amongst different participant groups. For instance, FGDs were used to stimulate discussions that helped contextualise community-level perceptions about FP and contraceptive use.

In the second round, IDIs were used to build on findings from the first round and to explore specific themes that had surfaced during the initial set of interviews and discussions. For all participants, topic guides included: socio-cultural and economic contexts, influence of contextual factors on FP, perceptions of the intervention—what worked and what did not work, and opinions about how perceptions of FP have changed. Additional topics for women included: their experience of FP and immunisation services, decision-making about contraceptive use and what influences use. Interviews with male community members also included: perceptions of FP/contraceptive use, what stops women from using contraceptives, community perceptions about women who use contraceptives. And interviews with implementers and intervention designers included: decision making about the integrated model and what makes the intervention work or not work.

### Adapted framework

**Fig. 1:**
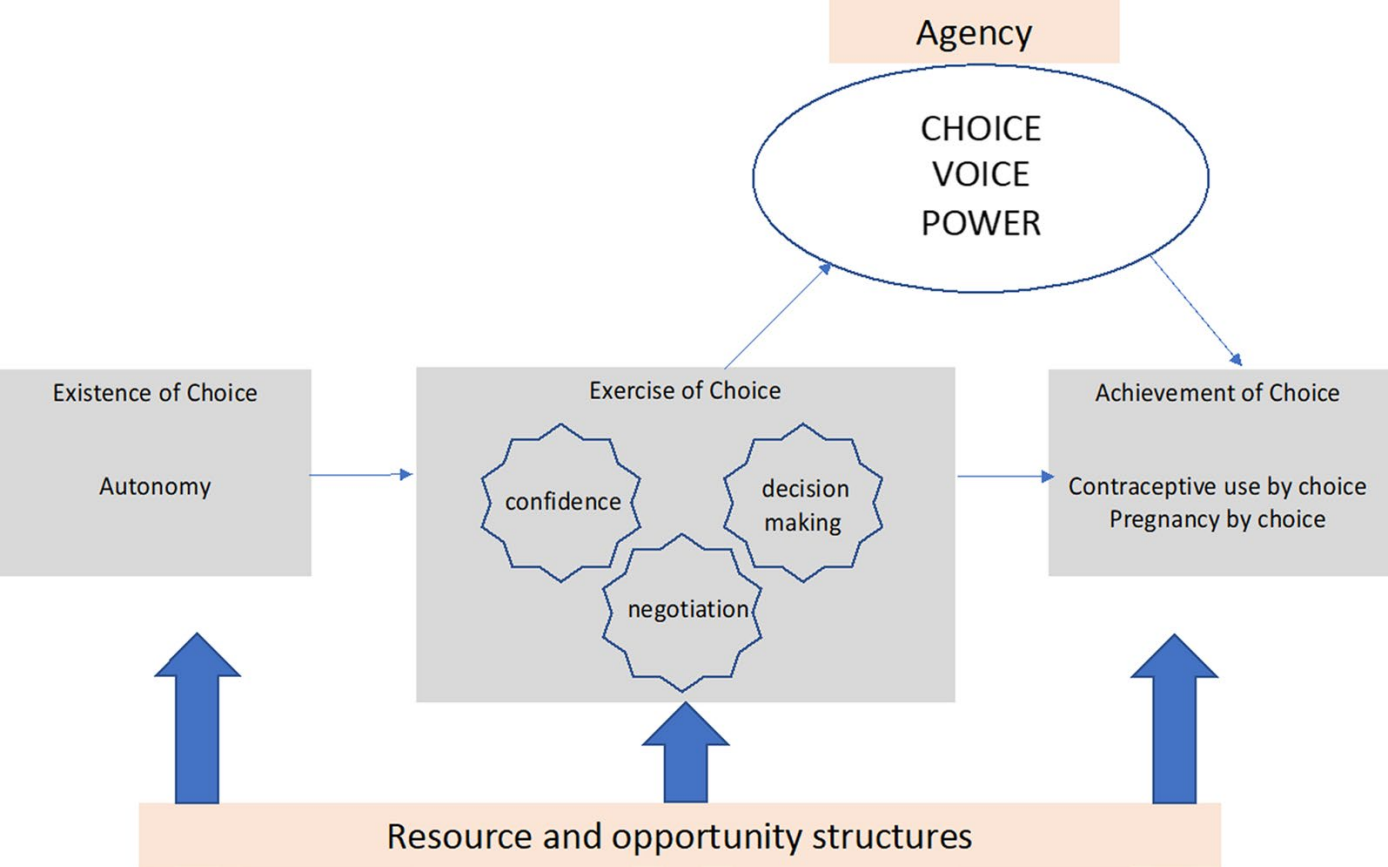
Adapted framework depicting the pathway involved in the decision to engage in covert contraceptive use

The SRH framework developed by Karp et al. [12] was adapted, expanded and used to organise and synthesise the findings of the thematic analysis (Fig. 1). In brief, Karp’s framework illustrates the pathway from *existence of choice*—where women recognise their reproductive preferences and set goals, to the *exercise of choice*—which includes self-efficacy, negotiation, and decision-making and is where women decide to act on their preferences. And finally, to the *achievement of choice*—where women act to achieve their reproductive goals*.* This process is underpinned by *resources and opportunity structures* that enable and constrain women’s choices and decisions. In Karp’s framework, ‘agency’ sits on the pathway between the *exercise of choice* and *achievement of choice* and is defined as ‘a woman’s ability to act in line with her choices and behave in line with achieved choices’.

Karp’s framework was selected for several reasons. First, it provided a useful structure to explore how women make decisions about contraceptive use in general and CCU specifically. Second, it enabled an examination of that decision-making process in the context of SRH empowerment. Here, SRH empowerment is defined as ‘*both a transformative process and an outcome, whereby individuals expand their capacity to make informed decisions about their reproductive lives, amplify their ability to participate meaningfully in public and private discussions related to sexuality, reproductive health and fertility, and act on their preferences to achieve desired reproductive outcomes, free from violence, retribution or fear’* [27]. Finally, the framework was developed, tested and refined in four culturally distinct settings in SSA, which is important for this analysis because empowerment is a deeply contextual construct.

Adaptations to, and expansion of, Karp’s framework were needed to accommodate the nuances of CCU. In the adapted framework, the term ‘self-efficacy’, referring to the attribute representing a woman’s confidence in voicing or acting on her reproductive preferences, was substituted by the term ‘confidence’. Although confidence is often considered to be synonymous with self-efficacy, here it is used to encapsulate both the feeling of being able to achieve a goal *and* a sense of self-worth. Agency is a key construct within other empowerment models and is referred to by some as the ‘active’ ingredient, whereby an individual makes a decision and carries out the necessary action with the aid of resources and structures at different levels [27]. But to understand *how* a woman embodies agency within the context of CCU, Karp’s framework was expanded to explore three distinct facets of agency: choice, voice and power [27, 28]. Choices can be real (when between two viable alternatives) or constrained (when either option is associated with high costs). Voice reflects a woman’s participation in discussions, where she can openly share her opinions and desires. Power *within* occurs when women ‘*perceive themselves as able and entitled to occupy decision making space*’ [29] and the power *to* is the ‘*realization that she can shape her circumstances to achieve a situation that is more favourable to her interests…’* [30].

### Data analysis & synthesis

The primary data analysis was conducted in line with the objectives of the evaluation and the data were coded and analysed following each round of data collection. SSI and FGD transcripts were imported into Nvivo 11.2 for coding and analysis. The data were coded around a framework based on the major themes from the topic guides and sub-themes were added inductively. IDI transcripts were imported into Nvivo 12 for coding and analysis. The coding framework included: context (cultural, religious, economic); decision-making about FP/contraceptive use—including barriers and facilitators to use. Additional themes and sub-themes were added inductively. For both data sets coding was done by one researcher and the thematic analysis was discussed with the research and implementing teams. Consensus was sought if disagreements arose. Transcripts were anonymised and were labelled only with the type of respondent to aid analysis.

For this study, data were then extracted from the thematic tables produced as part of both evaluations for each of the four countries. Relevant themes and sub-themes were extracted and mapped across the core components of the adapted framework and separated by study site. The components of the adapted framework included (1) *existence of choice*—social norms, culture, religious beliefs, reproductive preferences of women (including their desire to space or limit childbearing and how they want to achieve that—ie. by using contraceptives or natural methods) (2) *exercise of choice*—women’s decision-making, motivations for and consequences of CCU, discussions with husbands, gender dynamics and power within relationships, (3) *achievement of choice*—descriptions of personal or others CCU, (4) *agency*—choice, voice and power, and (5) *resources and opportunity structures*—health worker/implementer support for CCU, barrier and facilitators to FP services/contraceptives, side effects, integration with child immunisation services. Using illustrative quotes, and guided by the adapted framework, a narrative was then created to describe the decision-making process in which women use contraceptives covertly. A further synthesis of the data involved using the narrative findings to understand the role of agency in the decision to engage in CCU. Data from both time points were used, not as a temporal exploration of CCU, but rather because some themes were explored in greater depth in the second round of data collection and thus enabled a more thorough examination of the process. To ensure rigourous reporting, the methods and results presented in this study follow the Standards for Reporting Qualitative Research (SRQR) [31].

## Results

Qualitative data from 130 women (contraceptive users and non-users), 126 health providers, 55 community members and leaders, 40 male community members, 49 health administrators and intervention implementers from four countries were used to explore women’s decision-making around CCU (Table 2). The results are presented around the following thematic categories: (1) women exercise choice by using contraceptives covertly to reach their reproductive goals, (2) a transformational shift in women’s perception of *who* is entitled to make decisions about contraceptive use influences their decision to engage in CCU, and (3) CCU was sometimes perceived to challenge norms around contraceptive decision-making.

**Table 2:** SSIs, FGDs and IDIs by participant group and country

	Benin			Kenya			Malawi			Uganda			Total
	SSIs	FGDs	IDIs	SSIs	FGDs	IDIs	SSIs	FGDs	IDIs	SSIs	FGDs	IDIs	
Women	–	3 (20)	13		4 (43)	14	7	–	18	5	–	10	130
Health providers	6	–	15	5	3 (23)	13	3	4 (25)	8	14	–	14	126
Community members/leaders	–	–	8	2	2 (20)	7	2	–	5	3	–	8	55
Male community members		2 (10)	6	–	2 (15)	2	–	–	5	–	–	2	40
Health administrators/implementers	4	–	5	5	–	7	10	1 (3)	6	5	–	4	49
Total	87			156			92			65			400

FGDs (n) = total participants

### Using CCU to exercise choice and achieve reproductive goals

In the adapted framework, *exercise of choice* is comprised of three components: confidence, decision-making and negotiation. Women in this study engaged in CCU when they had the confidence to make their own decisions and were able to bypass the negotiations with their husbands, either initially or following previous failed negotiations.

#### Acquiring the confidence

Women from all settings described how they suffered the consequences of unplanned fertility, primarily in terms of the negative effects on their health and the economic challenges of supporting a large family. Some women spoke specifically of the pain of childbirth, while others spoke more broadly about wanting to avoid extreme poverty by limiting the number of children they have. Irrespective of the reason, women were clear that it was them who suffered the consequences whilst their husbands rarely considered the impact of frequent births and of providing for large families.

“Interviewer (I): Do you think women are required to talk to their male partners before deciding to start using it [FP]? Respondent (R): No. It is not an obligation. I: Why? R: Men hardly think of the consequences of short birth spacing.” Woman 10, Benin

“I just evaluated the status and nature of my poor family and concluded that family planning was the immediate remedy to bail me out of poverty and agony that I had already started to experience. My husband was negligent to care for us and I saw doom in our future…It was my own decision to join family planning after seeing the danger I was facing ahead of the family.” Woman 2, Uganda

Some women went further to acknowledge the injustice of not controlling their contraceptive autonomy despite bearing the consequences. Recognising the imbalance of this situation women concluded that they were morally justified in using contraceptives without telling their husband, which gave them the confidence to make the decision to engage in CCU.

“R: Personally, my husband didn’t know that I did it [use FP], for example. I would be the one to suffer if we had several young children and I was pregnant at the same time. My husband simply gets dressed and leaves the house. He doesn’t live in the same world as me. This is why I did it before telling him.” Women’s FGD 7, Benin

“R: they [husbands] just want you to have many kids; but I believe that’s not fair since it’s us women who experience the pain when giving birth while the duty of the husband is just to impregnate you; this is the reason women take FP without their husband’s knowledge…" Woman 9, Malawi

However, not all women acquired the confidence to engage in CCU. For some, the risks were too great, and the consequences were perceived as punishingly high. Health providers and women described situations where covert use led to domestic violence. There were also concerns about marriage breakdown resulting from women disobeying their husband.

“I: …does it really prevent them [women] from adopting a method? R: Yes, because there are some when they do it, their husband notices and dumps them.” Health worker 8, Benin

“R: …only that some women are not allowed by their husbands to use family planning methods. For instance, where I live, there are some men who beat up their wives once they hear that they went to take some family planning methods.” Woman 17, Malawi

#### The ultimate decision makers

Women who reported engaging in CCU explained that the choice to use contraceptives was theirs, even without their husband’s consent. Women frequently made the link between their deeper awareness of the negative consequences that arise as a result of not using contraceptives and as such felt they needed to make the decision to use contraceptives with or without their husband’s support.

“I: Is it compulsory for the woman to inform her husband before using a birth control method? R: Yes, if your husband is not in conflict with you. But if you want you can choose not to inform him.” Woman 13, Benin

“R: he [husband] doesn’t want [FP]… but for me as a woman who watches how my family is because he knows nothing about his family and that is why I take the difficult decisions and I also go without letting him know … he will not accept, my responsibility is also to practice without his knowledge.” Woman (peer influencer) 18, Uganda

Not all women shared this thinking or perceived this choice as available to them, for some once a husband said no to using contraceptives there were no alternatives. Whilst for other women joint decision-making on contraceptive was preferred and considered the best way to avoid conflict.

“I: and what if he [husband] refuses? R: then you just stay because you have no option…I: who makes decision about the number of children that one is supposed to have? R: it’s the husband…" Woman 5, Kenya

#### Bypassing the negotiations—the importance of resources and opportunity structures

In this study, women described the challenges when speaking about contraceptive use with their husbands. Some women described initial attempts to discuss contraceptive use with their husband but opted to avoid future negotiations once a refusal was voiced. Other women felt that speaking with their husband would be difficult, primarily because they believed or knew their husbands did not approve of contraceptive use. This reasoning was also acknowledged by men. Some women described using contraceptives covertly to start, suggesting that if they started using them their husbands may accept it in the future.

“I: What do you think makes women to use family planning methods without their husband knowledge? R: They think that if they consult their husbands, they [will be] denied, so they just do it behind them.” Male partner 9, Malawi

“R: For me, I will not inform my husband because we were talking once and he said that if a woman goes for family planning it is for her to enter prostitution. For that reason, when I am ready, I won’t say anything to my husband.” Woman 2, Benin

Once women had decided to take up a contraceptive covertly, various resources and support structures were needed to support that decision and keep the practice concealed from their husbands. This included supportive FP services that recognised a woman’s right to use contraceptives without permission from her husband and the availability of discreet methods. For some women, the integration of FP services with childhood immunisations provided a convenient reason to visit a health facility and receive a method, and helped facilitate covert use. Importantly, some providers felt that autonomous decision-making was not ideal and that women should make the decision about contraceptive use together with their husband to ‘keep the harmony’ at home. Consequently, there were women who described situations where providers stressed the importance of asking permission from their husband to use contraceptives.

“R: …we have those clients who have that fear of telling their partner that, am going for a family planning, so whenever they have their little baby, they carry for immunization but in their mind, they know am taking my baby to the immunization, but at the same time I will take family planning, yes.” Nurse 11, Kenya

“R. The couple should discuss, evaluate the family situation and agree family planning with informed consent. I. Why should a wife consent with the husband about entry to family planning? R. It is for family harmony and unanimous consent. The couple after jointly agreeing the family planning will begin planning on the next move to space children.” Community health worker 1, Uganda

Some participants in Benin acknowledged legislation that supported women’s rights to access FP services without permission from their husbands. In addition, the provision of free contraceptives was perceived to have enabled women to engage in CCU.

"I: Do you know whether there is such a law? R: Yes, I heard about that. It allows the woman who is exhausted from giving birth to adopt a family planning method without her husband's agreement in order to take a break in her household, and the man can do nothing against her.” Woman (group leader) 1, Benin

However, women still faced barriers when engaging in CCU. Side effects, such as spotting between cycles, made hiding contraceptive use difficult according to respondents. Health system challenges, such as stock outs of methods were also reported to have devastating effects on CCU.

"I have an elder relative who was also here, she has more than 5 children and as I am talking now she has conceived again because her husband restrains her from practicing family planning and she comes without husband’s consent to receive the method. Some weeks back we had run out of the injectables so she couldn’t travel long distances in fear that her husband might notice that she is practicing family planning, now she is pregnant.” Woman 2, Malawi

### The transformational shift in perceptions

Between *exercising* and *achieving* choice women need to act on their reproductive preferences. Women in this study faced constrained *choices* and had a limited *voice* in discussions about their reproductive aspirations. However, when women recognised the *power within* and the *power to—*an empowering transformational shift occurred that influenced their decision to engage in CCU. Specifically, this involved a shift in a woman’s perception of *who* is entitled to make decisions about contraceptive use.

#### Constrained choices

In the context of CCU, women had to choose between two undesirable options. Participants reported a lack of control in deciding the timing of births and the number of children. Women also shared their fears about, and experiences of, having covert use discovered. This indicates that their choice lies between a lack of control over their fertility or taking the risk of using contraceptives covertly. Women perceived both options as having potentially negative consequences.

“R: I am not the only wife of my husband, we are many. And I’d really like to use family planning. I already have four children and I would love to pause and concentrate on money making before I start over again, but my husband is not in that logic.” Woman 2, Benin

#### Limited voices

One of the key features of CCU is that women lack the ability to articulate their fertility preferences within their relationship, such as discussing their desired family size. Many health providers in this study emphasised the importance of providing FP services to women regardless of whether they had permission from their husbands. However, as previously noted, where providers stressed the importance of obtaining permission from the husband, women’s voices were again constrained. As such, women’s voices were limited to settings where health providers were supportive and where they were able to discuss their reproductive preferences and ask for help in achieving their goals.

“I: The first consent of course comes from the husband, if he says no… R: No, but not if this mother has brought the child for immunization, and she is willing, she is accepting the method. Why should I let this mother have to get consent from the husband?" Midwife 5, Uganda

“R: The health workers and also the people who came to talk to us, all said it’s important that we tell our husbands before using a method of FP. So, you tell your husband and he objects. And then we don’t have any choice.” Women’s FGD 5, Benin

#### Recognition of ‘power within’ and ‘power to’

In this study, when women recognised that they were entitled to occupy the decision-making space with regards to their fertility and realised that they could act to improve their situation**—**they felt justified in making the decision to use contraceptives covertly.

"…for some men, the woman is to give birth as many times as they want, even to the point of dying. They don’t mind. This is what some men think. When it comes to this category of men, they don’t approve their wives using a family planning method. Thus, women confronted with this situation, secretly adopt a family planning method behind closed doors…” Woman (group leader)1, Benin

Women shared other ways in which their decision to engage in CCU was a deliberate act of defiance**—**where they could exert control over a situation that did not serve them.

“R: For example, if the woman is being cheated by her husband, she may not allow her husband to impregnate her so she may start using family planning to punish the promiscuous husband.” Woman 12, Malawi

### Covert use in contrast: harmful consequences and challenging norms

In this study, there were women who reported that their covert use of contraceptives had helped to dispel the myths and misconceptions they felt blocked their husband’s approval of contraceptive use, such as infertility and disrupted sexual relations. For these women, being able to prove using contraceptives did not lead to feared outcomes was perceived to give women greater negotiating power. But the consequences of CCU were also highlighted by some health providers and women who noted that, when uncovered, the practice could lead to domestic issues and, in some cases, violence.

“I: What about the father of your children, he did not influence you to go for it? R: No, I decided on my own and after I had gone for it, he accepted it because he saw that it was good.” Woman 4, Kenya

"R: ….only that some women are not allowed by their husbands to use family planning methods. For instance, where I live, there are some men who beat up their wives once they hear that they went to take some family planning methods.” Woman 17, Malawi

Some health providers and implementers reflected on how CCU had catalysed opportunities to engage with husbands about FP and contraceptive use. This was perceived to be an important outcome of CCU as husbands often lacked information about contraception.

"…A man comes to the facility very annoyed, why is my wife on family planning? And yet, the wife wants family planning, but the husband does not… they called the wife and they counselled them together. They talked to him about all the family planning methods, and why the wife chose that method of the implant, and also about the side effects and how they can be managed… And the man went back excited, and also accepting this method…” Implementer 6, Uganda

## Discussion

To our knowledge, this is the first time a SRH empowerment framework has been used to examine the process through which women make the decision to engage in CCU. The findings suggest that the decision to engage in CCU is influenced by a shift in the woman’s perception of who is entitled to make decisions about contraceptive use. This transformation occurs as women recognise their ability to alter a situation that does not serve them and find the necessary moral justification to do so. This has important implications for FP programme implementers, including health providers, who seek to empower women through the provision of SRH services. Specifically, findings suggest that a woman-centred approach to service provision, one that respects the autonomy of women as decision-makers, can support those who choose to use contraceptives covertly. The recognition by implementers that resources and opportunity structures, such as steady stocks of discreet methods and effective side effect management, are key in enabling women to exercise choice is crucial.

At all study sites, women discussed the contextual factors that forced them or others to conceal contraceptive use. From discordant fertility preferences to male disapproval of contraceptive use, women acknowledged the hurdles faced when contemplating use. These barriers have been widely described in the literature [2, 32] and reflect the symptoms of patriarchal structures within which women make reproductive decisions [3]. In the context of SRH empowerment these external factors, combined with women’s internal motivations, shape fertility desires and ambitions [6, 12]. Women engaged in CCU when they believed their fertility choices were not supported within their relationship. However, in contexts where male disapproval towards contraceptive use is common, some women avoid discussing contraceptive use with their husbands due to the assumption they will be denied permission [8] and, in some cases, disclosure of covert use could improve communication about contraceptive use [11].

In this study, women who used contraceptives covertly to *exercise choice*, described a sense of entitlement because they suffered the consequences of short birth intervals and caring for many children, as a key driver of their autonomous decision-making. And whilst rationales for why women engage in CCU have been explored previously [8, 9, 11], identifying this justification contributes to a deeper understanding of how women gained the confidence to use contraceptives covertly. This justification was also central to women recognising the power-within, which is at the heart of the transformational shift described in this paper. Both the power-within and confidence relate to the concept of critical consciousness**—**where women begin to question inequalities and power relations and assert their sense of entitlement [30]. The acknowledgment from women in this study that the decision to use contraception was rightfully theirs, reflects critical consciousness around this facet of reproductive health and rights. This recognition is crucial in building the collective power of women and catalysing wider social change where women can assert control over their reproductive faculties [28].

The way in which CCU is understood, particularly within the context of SRH empowerment, has important implications for how FP programme designers and implementers deal with this practice at the point of care. More broadly, CCU encapsulates women’s reproductive disempowerment**—**it illustrates how patriarchal systems and societies constrain a woman’s ability to voice her preferences and choose her reproductive destiny free from fear. But a more granular exploration of the process through which women choose to use contraceptives covertly, reveals a nuanced picture, where a transformational shift is obscured by the oppressive structures that force her decision to be hidden. The experiences shared by women in this study indicate that CCU can occur because of a significant shift in a woman’s perceived right to self-determination with regards to her fertility. And yet despite this, the decision to engage in CCU can have potentially devastating consequences, such as domestic violence and marriage breakdown [8]. In addition, as a gender accommodating practice CCU does little to address the barriers that constrain women’s reproductive choices. However, it could play a role in disrupting gender norms and challenging the existing inequalities around who makes decisions about contraceptive use. These tensions represent significant challenges when delivering FP services. Kibira et al*.* [11] similarly concluded that while it is critical to continue working to shift male perceptions of contraceptive use and promote healthy discussion among couples, the experiences shared by participants in this study highlight the need for FP services to offer a safe space for women to express their reproductive preferences and make autonomous decisions if needed.

This study underscores the importance of resource and opportunity structures in the context of CCU. With a deeper understanding of the transformational shift that influences the decision to engage in CCU, health providers can indirectly support women’s contraceptive choices by acknowledging them as the decision-maker. But finding this balance in a context where joint decision-making on contraceptive use may be preferred can be challenging. A study examining the role of health providers in women’s decision making about contraceptive use in Ethiopia found that women were uncomfortable when asked if they had their husband’s permission prior to being issued a contraceptive method [33]. This line of questioning, even if well-intentioned, can undermine a women’s perception that she is entitled to make decisions about contraceptive use herself. Even when legislation supports a woman’s right to make autonomous decisions about contraceptive use, it is the attitudes and choices made by health providers that will ultimately have the greatest impact on the delivery of FP services that respect women’s reproductive preferences. Women interviewed discussed a number of additional challenges when using contraceptives covertly, such as stock outs of discreet methods and visible side effects, a finding echoed by studies conducted elsewhere in SSA [11]. It is critical for health providers to balance supporting women to make choices based on their reproductive desires, with helping them to minimise the risks associated with CCU, including screening for domestic violence. Effective management of side effects is a key area where health providers can assist women in concealing their contraceptive use. In this study, integrating FP services with childhood immunisations emerged as an important way through which women were able to access contraceptives without having to tell their husbands. However, very careful monitoring must be in place to ensure integrating FP services in general, and CCU in particular, do not harm the immunisation programme.

Overall, the adapted framework used in this study provided the opportunity to expand on the concept of ‘agency’. This facilitated an exploration of how specific constructs of agency (choice, voice and power) shape women’s ‘decision to act’ in the context of CCU. By using the basic building blocks of agency, a transformational shift was identified that begins when women start to question the inequalities that constrain their reproductive choices and decisions. This proved useful in exploring the process of SRH empowerment at the individual level. To adequately capture the recognition of injustice that underpins a woman’s decision to engage in CCU, the term self efficacy was replaced with confidence in the adapted framework. This paper provides insights on how CCU could be challenging gender norms around contraceptive use, but further research is required. For instance, a better understanding of how women shift their husband’s perceptions of contraceptives by using them covertly would be very useful.

### Limitations

This study has some important limitations. The demographic data of participants was not captured, which limits the contextual description of the respondents. Participants were selected as part of an evaluation on integrated FP and childhood immunisation services and as such, the primary purpose for data collection was not to explore CCU. Thereby, the full range of CCU experiences and perspectives are likely not reflected in these findings as the researchers did not set out to explore the phenomenon of CCU but rather followed the thematic patterns as they emerged from the data. However, this approach may have inadvertently mitigated the potential for social desirability bias as most respondents discussed their experience with CCU spontaneously, rather than being asked specifically about the topic. It is therefore unlikely that responses were provided to satisfy the researcher. This study is strengthened by the inclusion of a range of participants from sites in four countries, though the generalisability of findings to settings outside the study is limited. However, the use of an established framework to support the analysis helps with abstraction of findings, and increases the potential for the transferability of the findings to other similar contexts. Furthermore, although the data were coded and analysed by one researcher, the findings were discussed and scrutinised by all members of the research team, including validation by the local implementers.

## Conclusion

This study confirmed that CCU is an important way in which women exercise choice and achieve their reproductive goals and highlights the cruical role of health providers in acknowledging women as decision-makers. Women engaged in CCU because they felt confident to make their own decisions and were able to bypass negotiations with their husbands, often because they could access FP services and use a contraceptive method discreetly. Health providers play a critical role in supporting women by listening to their reproductive preferences and helping them to overcome the challenges of using contraceptives covertly, such as effective management of side effects. Although CCU is not an easy option, for some women it follows a transformational shift in their perception of who is entitled to make the decision about contraceptive use. These findings suggest that women engage in CCU when they recognise that they are entitled to make decisions that align with their reproductive goals and that they can change a situation that does not serve them.

## Data Availability

The datasets generated and/or analyzed during the current study are not publicly available in order to maintain the anonymity of the participants. The data can be made available from the corresponding author on reasonable request.
